# The Clinical Significance of Mesenteric Lymphocytes in Human Colorectal Cancer

**DOI:** 10.3389/fonc.2021.685577

**Published:** 2021-09-16

**Authors:** Zi-Xin Wu, Fei Wang, Liang Li, Yuan Yao, Jie Long, Qing-Qing Luo, Zhi-Bin Zhao, Wang-Lin Li, Jie Cao, Zhe-Xiong Lian

**Affiliations:** ^1^Department of Colorectal Surgery, The Second Affiliated Hospital of South China University of Technology, Guangzhou, China; ^2^School of Medicine, South China University of Technology, Guangzhou, China; ^3^Guangdong Provincial People’s Hospital, Guangdong Academy of Medical Sciences, Guangzhou, China; ^4^Department of Thoracic Surgery, Guangdong Provincial People’s Hospital, Guangdong Academy of Medical Sciences, Guangzhou, China

**Keywords:** colorectal cancer, clinical significance, immune microenvironment, lymphocyte, mesentery

## Abstract

**Objective:**

The mesentery is a potential site of residual tumor in patients with colorectal cancer (CRC). However, the mesenteric immune microenvironment remains unclear. In this study, we investigated the immune landscape of the mesentery, particularly the role of lymphocytes and its association with the clinicopathological characteristics of CRC.

**Methods:**

Flow cytometry was used to detect lymphocytes in the paired mesenteric tissue specimens adjacent to the colorectal tumors and normal mesenteric tissue specimens 10 cm away from the colorectal tumor edge and preoperative peripheral blood samples obtained from patients with CRC who underwent surgery. T-distributed stochastic neighbor embedding was utilized to analyze multiparameter flow cytometry data. Multiplex immunohistochemistry was performed to evaluate T cells subsets in the paired mesentery adjacent to the colorectal tumors and normal mesentery. The Fisher’s exact test and non-parametric Wilcoxon’s matched-pairs tests were used for statistical analysis. The non-parametric Mann-Whitney U test was used to determine associations between percentage data and clinical parameters of patients with CRC.

**Results:**

We found that immune cells in the normal mesentery were mainly of lymphoid lineage. Compared with peripheral blood, the normal mesentery showed decreased NK cells and the CD4/CD8 ratio and increased CD3^+^ CD56^+^, memory CD4^+^ T, memory CD8^+^ T, CD4^+^ tissue-resident memory T (TRM), and CD8^+^ TRM cells. Compared with the normal mesentery, the mesentery adjacent to the colorectal tumor showed increased B and regulatory T cells and decreased NK, CD3^+^ CD56^+^, CD4^+^ TRM, and CD8^+^ TRM cells. Moreover, memory CD8^+^ T cells and plasmablasts are negatively correlated with the depth of invasion of CRC. Increased memory CD4^+^ T cells are associated with distant metastasis of CRC and high preoperative serum carcinoembryonic antigen levels.

**Conclusion:**

The mesentery shows a specific immune microenvironment, which differs from that observed in peripheral blood. CRC can alter the mesenteric immune response to promote tumor progression.

## Introduction

Colorectal cancer (CRC) is one of the most common malignant tumors, with an estimated 1.8 million new cases worldwide in 2018 and ranks third with regard to incidence and second with regard to mortality (1). Studies have shown that postoperative recurrence was associated with cancer-related death in most cases (2–4), and mortality rates were 3.47-fold higher in patients with CRC recurrence than in those who were recurrence-free (5). An intraoperative residual tumor is one of the main contributors to postoperative local CRC recurrence (6). The mesentery is a continuous membrane that attaches the intestines to the abdominal wall and is considered a potential site of residual tumor (7, 8). Metastatic cancer cells were detected in the colorectal mesentery in approximately 20% of patients with CRC, who underwent radical surgery (9, 10), and patients with mesenteric metastasis tend to show poor cumulative survival rates (11, 12).

Currently, the mesentery is classified as an organ that contains not only adipocytes but also blood vessels, lymphatics, nerve tissues, immune cells, and connective tissue matrix (13). Reportedly, mesenteric immune cells play a significant role in the pathogenesis and disease progression of intestinal diseases, particularly Crohn’s disease (14–17). Under steady-state conditions, the mesentery shows large numbers of anti-inflammatory cells, including M2 macrophages and regulatory T (Treg) cells (17). However, following intestinal inflammation, most immune cells detected in the mesenteric adipose tissue comprise pro-inflammatory cells, such as M1 macrophages (17). Moreover, compared with controls, CD68- and CD163-positive cells were shown to be significantly increased in the mesentery in patients with CRC (18). However, lymphocyte composition and phenotype in the mesenteric immune microenvironment remain unclear.

In this study, we investigated the lymphocyte landscape of the mesentery and the association between lymphocytic infiltration of the mesentery adjacent to the tumors and clinicopathological characteristics of patients with CRC. We observed that colorectal tumors can alter the lymphocyte phenotype of the mesentery. Memory CD8^+^ T cells and plasmablasts were negatively correlated with the depth of invasion of CRC, and memory CD4^+^ T cells were associated with distant metastasis of CRC, which highlight the role of the mesentery in CRC.

## Material And Methods

### Specimen Acquisition

We obtained 32 paired mesenteric tissue specimens adjacent to the colorectal tumors (MES T) and normal mesenteric tissue specimens (MES N) 10 cm away from the colorectal tumor edge in patients with CRC who underwent surgery between 2018 and 2020 at the Department of General Surgery, Guangzhou Digestive Disease Center, Guangzhou First People’s Hospital (Guangzhou, Guangdong, China). Peripheral blood (PB) samples were obtained from 13 of these patients preoperatively. Demographic and clinicopathological characteristics of 32 patients with CRC are shown in **Table 1**. Patients with a history of inflammatory bowel disease were excluded. The study protocol was approved by the Guangzhou First People’s Hospital Ethics Review Committee, and written informed consent was obtained from all patients. All specimens were anonymized.

**Table 1:** Demographics and clinicopathologic characteristics of patients.

Characteristics	n (%)
**No. patients**	32
**Gender**	
Male	17 (53.1)
Female	15 (46.9)
**Age (years)**	
Mean ± SD	61.2 ± 13.5
<55	9 (28.1)
≥55	23 (71.9)
**Tumor location**	
Ascending colon	10 (31.3)
Transverse colon	5 (15.6)
Descending colon	2 (6.3)
Sigmoid colon	11 (34.4)
Rectum	4 (12.5)
**Maximum tumor diameter (cm)**	
<4	13 (40.6)
≥4	19 (59.4)
**T stage**	
2	3 (9.4)
3	14 (43.8)
4	15 (46.9)
**N stage**	
0	17 (53.1)
1	9 (28.1)
2	6 (18.8)
**M stage**	
0	17 (53.1)
1	5 (15.6)
x	10 (31.3)
**Preoperative serum** **CEA level (ng/mL)**	
<5	21 (65.6)
≥5	11 (34.4)

SD, standard deviation; CEA, carcinoembryonic antigen.

### Tissue Digestion and Cell Isolation

Mesenteric tissues were dissected, and lymph nodes and blood vessels were carefully removed. The remaining mesenteric tissues were washed with 1× phosphate-buffered saline containing 0.2% bovine serum albumin and cut into small fragments in a petri dish. Subsequently, tissues were treated with a mixture containing 10 mL of Dulbecco’s Modified Eagle Medium (DMEM)/F12 1:1 (1×) (HyClone, Logan, UT, USA) mixed with 1 mg/mL of collagenase II (Sigma, St. Louis, MO) for 30 min at 37°C in a shaking incubator at 200 rpm. Cell suspensions were passed through a nylon mesh (74 μm) and centrifuged at 450 ×g for 5 min at room temperature. Then, cells were mixed with 2 mL red blood cell lysis buffer (Beyotime, Shanghai, China) for 10 min at 4°C for depletion of red blood cells, and centrifuged at 450 ×g for 5 min at room temperature. PB samples (2 mL) were collected in a tube containing ethylenediaminetetraacetic acid, and plasma was removed after centrifugation at 450 ×g for 5 min. An equal volume of physiological saline was added to resuspend the cell pellet. Cell suspensions were transferred to a new tube containing 3 mL Ficoll (Lymphoprep, Alere Technologies AS, Oslo, Norway) for density centrifugation according to the manufacturer’s instructions. Mononuclear cells (MNCs) were collected at the interface after density centrifugation. Cells were counted after they were treated with trypan blue stain.

### Flow Cytometry

Single-cell suspensions were initially incubated with the human Fc receptor blocking reagent and stained for 20 min at 4°C using a cocktail of fluorochrome-conjugated antibodies. Antibodies included anti-TCRVα7.2 PE/Cy7 (3C10), anti-CD161 PE/Dazzle 594 (HP-3G10), anti-CD25 PE/Cy5 (BC96), anti-CD69 PE (FN50), anti-CD56 BV605 (5.1H11), anti-CD45RO BV421 (UCHL1), anti-CD45RA BV510 (HI100), anti-CD19 BV785 (HIB19), anti-CD3 BV711 (OKT3), anti-CD103 APC (Ber-ACT8), anti-CD8α Alexa Fluor 700 (HIT8a), anti-CD45 APC/Cy7 (HI30), anti-CD127 BUV737 (HIL-7R-M21), anti-CD4 BUV563 (SK3), anti-CD38 FITC (HIT2), anti-immunoglobulin (Ig)A PE (polyclonal), anti-IgG BV421 (G18-145), anti-IgD APC (IA6-2), anti-IgM Alexa Fluor 700 (MHM-88), and anti-CD19 BUV563 (SJ25C1). All antibodies were purchased from BioLegend, except for anti-CD127 BUV737 (BD Biosciences), anti-CD4 BUV563 (BD Biosciences), anti-IgG BV421 (BD Biosciences), anti-CD19 BUV563 (BD Biosciences), and anti-IgA PE (SouthernBiotech). Following treatment with surface marker stains, cells were fixed and permeabilized using the Foxp3/Transcription Factor Staining Buffer Set (eBioscience, San Diego, CA, USA) to detect the transcription factor Foxp3 (anti-Foxp3 PE, 206D, BioLegend). Data were obtained using the BD LSRFortessa™ flow cytometer (BD Biosciences, San Jose, CA, USA) and analyzed using FlowJo software (BD Biosciences, San Jose, CA, USA).

### T-Distributed Stochastic Neighbor Embedding Analysis of Multiparameter Flow Cytometry Data

We gated the CD45^+^ MNCs in the FlowJo software and exported the expression matrices of three different samples from peripheral blood mononuclear cells (PBMCs), MES N, and the MES T to perform t-distributed stochastic neighbor embedding (t-SNE) analysis of multiparameter flow cytometry data. Thereafter, we randomly selected 8400 cells from each matrices and merged these into a single matrix in the R software (version 3.5.3). We normalized the matrix by channel using a centered log ratio transformation method embedded in the NormalizeData function of the Seurat package (version 3.1.5). The t-SNE algorithm was run with the normalized expression matrix using the RunTSNE function of the Seurat package. Dimensionality reduction of 4300 CD45^+^ CD3^+^ CD56^-^ MNCs and 2250 CD45^+^ CD19^+^ MNCs from PBMCs, MES N, and MES T, respectively, was performed in a similar manner.

### Multiplex Immunohistochemistry Staining

PANO 7-plex IHC kit (TSA-RM) (Panovue, 0004100100) was used for multiplex immunohistochemistry (mIHC) staining. 8 paired formalin-fixed paraffin-embedded (FFPE) MES N and MES T slides from patients with CRC were dewaxed and rehydrated. After antigen retrieval, slides were blocked with goat serum at room temperature (RT) for 20 min. The primary antibody, CD69 (1:500, ab233396, Abcam) was incubated overnight at 4°C. Slides were washed and incubated with horseradish peroxidase-conjugated secondary antibody (Panovue, 0004100100) at RT for 10 min. Finally, tyramide signal amplification (TSA) dye620 (1:100) was applied for 10 min after washed. The second antibody, CD4 (1:500, ab133616, Abcam) was incubated at RT for 2 h. And then, TSA dye690 (1:100) was applied. The third antibody, Foxp3 (1:100, 98377, CST) was incubated overnight at 4°C, and TSA dye570 (1:100) was applied. The last antibody, CD8α (1:400, 70306, CST) was incubated at RT for 2 h, and TSA dye520 (1:100) was applied. Nuclei were stained with DAPI (Beyotime, C1006) at RT for 5 min. Slides were imaged and scanned using a Vectra Polaris multispectral imaging system (Akoya Biosciences), and multispectral images were acquired using Phenochart software, version 1.0.12 (Akoya Biosciences) to unmix and remove autofluorescence. And slides were imported into HALO software version 3.2 (Indica Labs, Corrales, CA) for all subsequent steps, including annotation, training, and classification of multispectral slides.

### Statistical Analysis

Data are presented as mean ± standard deviation. Statistical differences were determined using the Fisher’s exact test and non-parametric Wilcoxon’s matched-pairs tests. The non-parametric Mann-Whitney U test was used to determine the associations between percentage data and clinical parameters. All analyses were performed using the GraphPad PRISM software, version 8 (GraphPad Software, San Diego, CA), SPSS software, version 25.0 (SPSS Inc., Chicago, USA), and the R software, version 3.5.3. All p values were two-sided, and p<0.05 was considered statistically significant.

## Results

### Demographics and Clinicopathological Characteristics

The study included 32 patients (17 men and 15 women) with CRC (mean age 61.2 years) (**Table 1**). Notably, 23 patients were aged ≥55 years and 9 were aged <55 years. CRC lesions were located at the following sites: ascending colon (10 cases), transverse colon (5 cases), descending colon (2 cases), sigmoid colon (11 cases), and rectum (4 cases). The maximum tumor diameter was ≥4 cm in 19 and <4 cm in 13 patients, respectively. The 8^th^ edition of the American Joint Committee on Cancer tumor, node, and metastasis (TNM) staging system was used in our study. The distribution of tumors based on the T stage was as follows: T2 stage (3 patients), T3 stage (14), and T4 stage (15). Distribution of tumors per the N stage was as follows: N0 stage (17 patients), N1 stage (9), and N2 stage (6). The distribution of tumors per the M stage was as follows: M0 stage (17 patients), M1 stage (5), and Mx stage (10). The preoperative serum carcinoembryonic antigen (CEA) levels were ≥5 ng/mL and <5 ng/mL in 11 and 21 patients, respectively. We observed no statistical differences in gender and age (≥55 years and <55 years) between patients with CRC showing different TNM stages (T2-3 stages *vs.*T4 stage, N0 stage *vs.* N1-2 stages, M0 stage *vs.* M1 stage) and preoperative serum CEA levels (≥5 ng/mL and <5 ng/mL) (**Table S1**).

### A Specific Immune Microenvironment Exists in the Mesentery

**Figure 1:**
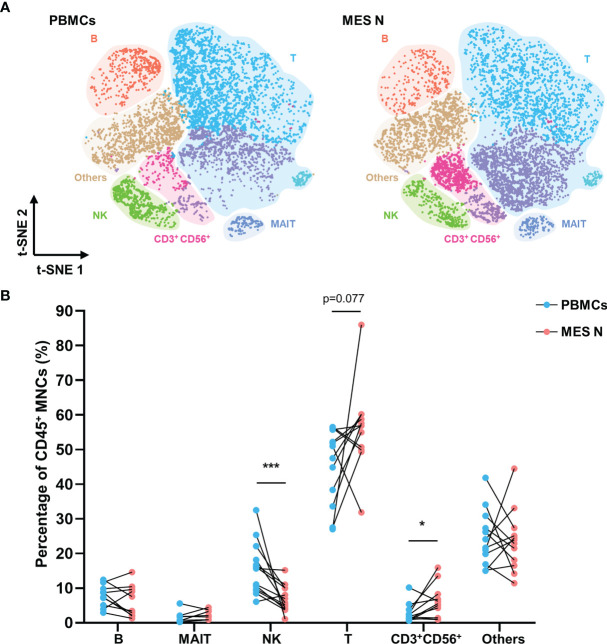
T-SNE plot and percentages of CD45^+^ MNCs in the PBMCs and MES N. **(A)** T-SNE plot of CD45^+^ MNCs in the PBMCs and MES N with main subclusters indicated. **(B)** Percentages of B cells, MAIT cells, NK cells, T cells, CD3^+^ CD56^+^ cells, and other cells (monocytes/macrophages and dendritic cells) in the PBMCs and MES N. (*p < 0.05; ***p < 0.001).

We identified six immune cell clusters among the CD45^+^ MNCs, including mucosal associated invariant T (MAIT) cells, T cells, NK cells, CD3^+^ CD56^+^ cells, B cells, and other cells (monocytes/macrophages and dendritic cells) (**Figure S1A**). MAIT cells, T cells, NK cells, and B cells were identified as CD3^+^ TCRVα7.2^+^ CD161^+^, CD3^+^ CD56^-^, CD3^-^ CD56^+^, and CD19^+^, respectively (**Figure S1B**). Based on the t-SNE plot of CD45^+^ MNCs, PBMCs and MES N differed significantly with regard to the compositions of T cells, NK cells, CD3^+^ CD56^+^ cells, and B cells (**Figure 1A**). Flow cytometry analysis showed that the percentage of NK cells was significantly lower in the MES N than in the PBMCs, and the percentage of CD3^+^ CD56^+^ cells was significantly higher than those of PBMCs (**Figures 1B** and **S2**).

**Figure 2:**
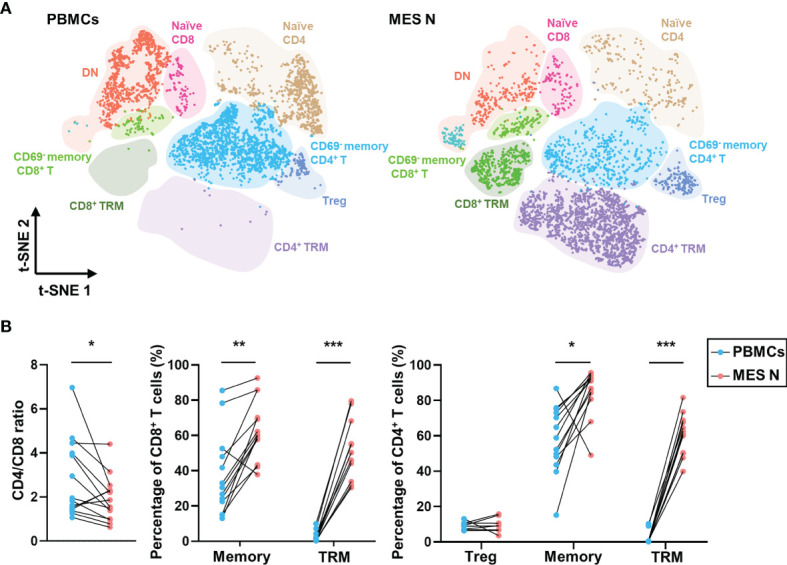
T-SNE plot and percentages of T cells in the PBMCs and MES N. **(A)** T-SNE plot of T cells in the PBMCs and MES N with main subclusters indicated. **(B)** CD4/CD8 ratio, percentages of memory CD8^+^ T cells, CD8^+^ TRM cells, Treg cells, memory CD4^+^ T cells, and CD4^+^ TRM cells in the PBMCs and MES N. (*p < 0.05; **p < 0.01; ***p < 0.001).

We categorized T cells into subclusters and identified each subcluster using several T cell-specific surface markers including CD4, CD8, CD45RO, CD45RA, CD69, CD103, CD25, and CD127 (**Figures S1C, D**). Memory CD4^+^ T cells, naïve CD4^+^ T cells, memory CD8^+^ T cells, naïve CD8^+^ T cells, and double-negative (DN) cells were identified as CD4^+^ CD45RO^+^, CD4^+^ CD45RA^+^, CD8^+^ CD45RO^+^, CD8^+^ CD45RA^+^, and CD4^-^ CD8^-^, respectively, based on expression levels of these specific markers. Treg cells were defined as those showing CD4^+^ CD25^+^ CD127^-^ expression (**Figure S1D**). Studies have reported that CD69 expression can indicate early activation of T cells, as well as their retention in tissues (19, 20) and that tissue-resident memory T (TRM) cells manifest upregulated CD69 expression, maintaining tissue resident and cytotoxic features (21–24). CD8^+^ CD69^+^ T cells have been shown to be more cytotoxic (25). In our study, CD4^+^ TRM and CD8^+^ TRM cells were phenotypically defined as those showing CD4^+^ CD45RO^+^ CD69^+^ and CD8^+^ CD45RO^+^ CD69^+^ expression, respectively (**Figure S1D**). As shown in **Figure 2A**, CD69 was expressed in T cells from the MES N but rarely in those from PBMCs. The CD4/CD8 ratio was significantly lower in the MES N than in the PBMCs; however, the percentages of memory CD8^+^ T cells, CD8^+^ TRM cells, memory CD4^+^ T cells, and CD4^+^ TRM cells were significantly higher in the MES N (**Figures 2B** and **S2**).

Based on CD38 and IgD expression levels (26), B cells can be clustered into plasmablasts (CD38^+^ IgD^-^), unswitched B cells (CD38^-^ IgD^+^), and memory B cells (CD38^-^ IgD^-^) (**Figures S1E, F** and **S4A**). B cells can undergo class-switch recombination from initial expression of membrane-bound IgM and IgD to membrane-bound IgG, IgA, or IgE in response to antigen stimulation (27, 28). As shown in the t-SNE plot of B cells, no significant differences were observed between PBMCs and MES N with regard to the compositions of plasmablasts, unswitched B cells, and memory B cells (**Figure S5A**). However, differences can be observed in the expression levels of IgM, IgA, and IgG in B cells between PBMCs and MES N. Flow cytometry analysis showed no statistically significant differences in B cell subclusters between PBMCs and the MES N; however, the percentages of unswitched B cells and IgM^+^ unswitched B cells in the MES N showed a downward trend, and the percentage of IgA^+^ memory B cells in the MES N showed an upward trend (**Figures S4A** and **S5B**). Therefore, our data confirm the existence of a specific mesenteric immune microenvironment, which differs from that observed in the PB.

### Decreased Tissue-Resident Memory T Cells and Increased Regulatory T Cells in the Mesentery Adjacent to the Colorectal Tumor

**Figure 3:**
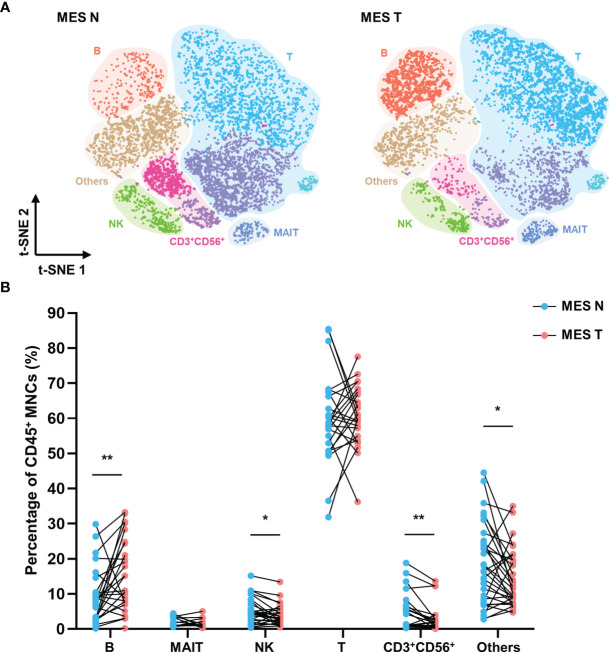
T-SNE plot and percentages of CD45^+^ MNCs in the MES N and MES T **(A)** T-SNE plot of CD45^+^ MNCs in the MES N and MES T with main subclusters indicated. **(B)** Percentages of B cells, MAIT cells, NK cells, T cells, CD3^+^ CD56^+^ cells, and other cells (monocytes/macrophages and dendritic cells) in the MES N and MES T. (*p < 0.05; **p < 0.01).

In view of its function as a conduit of intestinal blood and lymph, the mesentery is known to be involved in a variety of intestinal diseases. However, the effects of colorectal tumors on the mesenteric immune phenotype remain unclear. We investigated the immune landscape of the MES T. The t-SNE plot showed that the MES N and MES T differed with regard to the compositions of T cells, NK cells, CD3^+^ CD56^+^ cells, and B cells (**Figure 3A**). Flow cytometry data analysis revealed that the percentage of B cells was significantly higher and the percentages of NK cells, CD3^+^ CD56^+^ cells, and other cells (monocytes/macrophages and dendritic cells) were significantly lower in the MES T than in the MES N (**Figure 3B**).

**Figure 4:**
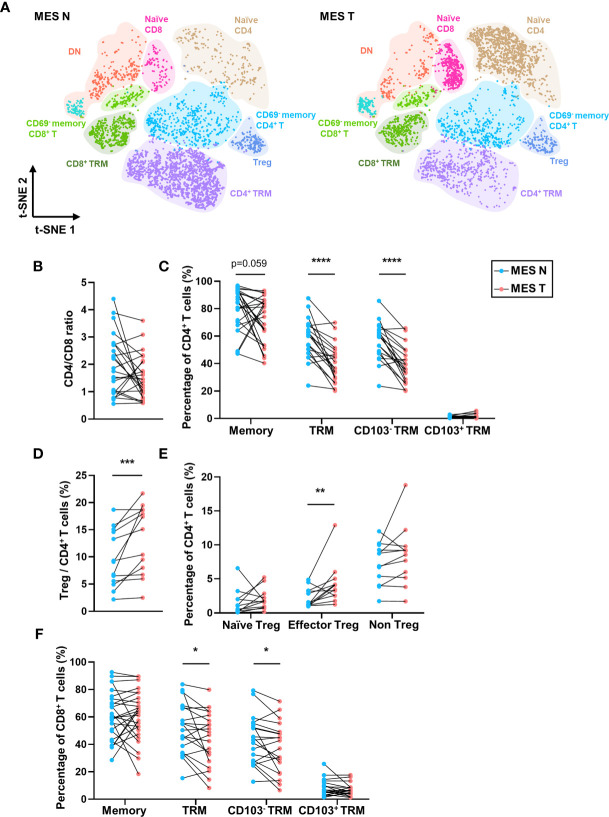
T-SNE plot and percentages of T cells in the MES N and MES T. **(A)** T-SNE plot of T cells in the MES N and MES T with main subclusters indicated. **(B)** CD4/CD8 ratio in the MES N and MES T. **(C)** Percentages of memory CD4^+^ T cells, CD4^+^ TRM cells, CD4^+^ CD103^-^ TRM cells, and CD4^+^ CD103^+^ TRM cells in the MES N and MES T. **(D)** Percentage of Treg cells in the MES N and MES T. **(E)** Percentages of naïve Treg cells, effector Treg cells, and non Treg cells in the MES N and MES T. **(F)** Percentages of memory CD8^+^ T cells, CD8^+^ TRM cells, CD8^+^ CD103^-^ TRM cells, and CD8^+^ CD103^+^ TRM cells in the MES N and MES T. (*p < 0.05; **p < 0.01; ***p < 0.001; ****p < 0.0001).

As shown in **Figure 4A**, we identified different T cell clusters in the MES N and MES T and observed that CD45RO and CD69 expression levels were significantly low in T cells in the MES T. Compared with the MES N, the percentage of memory CD4^+^ T cells showed a downward trend in the MES T, and the percentage of CD4^+^ TRM cells, particularly the CD4^+^ CD103^-^ TRM cells was significantly decreased in the MES T (**Figures 4B, C**). Moreover, the percentage of Treg cells was significantly higher in the MES T than in the MES N (**Figure 4D**). Reportedly, Foxp3^+^ Treg cells can be categorized into three subclusters (29), including naïve/resting Treg cells (Foxp3^lo^ CD45RA^+^), effector/activated Treg cells (Foxp3^hi^ CD45RA^-^), and non Treg cells (Foxp3^lo^ CD45RA^-^) (**Figure S4B**). Treg cells, particularly effector Treg cells induce marked immunosuppression, including suppression of T cell proliferation, cytokine production, and anti-tumor activity (29, 30). We observed that effector Treg cells were enriched in the MES T (**Figure 4E**). Additionally, CD8^+^ TRM cells, specifically CD8^+^ CD103^-^ TRM cells were significantly decreased in the MES T (**Figure 4F**). Furthermore, the percentage of Treg (Foxp3^+^ CD4^+^) cells was upregulated and the percentage of CD69^+^ CD4^+^ cells was downregulated in the MES T by mIHC (**Figures S3A–C**). However, the percentage of CD69^+^ CD8^+^ cells did not change in the MES T (**Figures S3A–C**). Moreover, although the t-SNE plot of B cells in the MES N and MES T did not significantly differ between B cell subclusters (**Figure S6A**), the percentages of unswitched B cells and IgM^+^ unswitched B cells in the MES T showed an upward trend (**Figure S6B**).

The reduced percentage of TRM cells and increased percentage of Treg cells in the MES T indicate poor anti-tumor immunity in the MES T. Therefore, it can be concluded that in addition to functioning as connective tissue that supports the intestine, the mesentery is an important component of the tumor microenvironment.

### Memory CD8^+^ T Cells and Plasmablasts Are Negatively Correlated With the Depth of Invasion of Colorectal Cancer, and Increased Memory CD4^+^ T Cells Are Associated With Distant Metastasis of Colorectal Cancer

**Figure 5:**
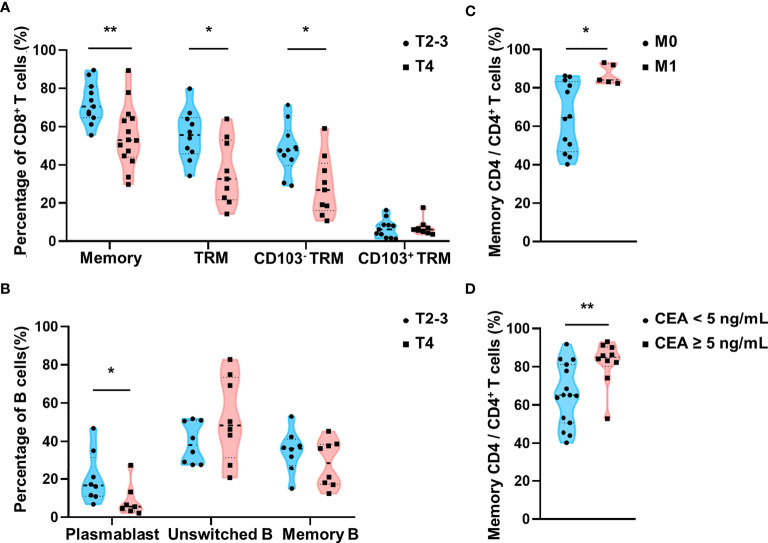
Relationship between lymphocytes in the MES T and TNM stages and preoperative serum CEA levels. **(A)** Comparison of the percentages of memory CD8^+^ T cells, CD8^+^ TRM cells, CD8^+^ CD103^-^ TRM cells, and CD8^+^ CD103^+^ TRM cells in the MES T between patients with T2-3 stage CRC and T4 stage CRC. **(B)** Comparison of the percentages of plasmablasts, unswitched B cells, and memory B cells in the MES T between patients with T2-3 stage CRC and T4 stage CRC. **(C)** Comparison of the percentage of memory CD4^+^ T cells in the MES T between patients with M0 stage CRC and M1 stage CRC. **(D)** Comparison of the percentage of memory CD4^+^ T cells in the MES T between patients with CRC showing different preoperative serum CEA levels (<5ng/mL and ≥5ng/mL). (mean ± SD; *p < 0.05; **p < 0.01).

We investigated the association between lymphocytes in the MES T and the tumor stage in 32 patients with CRC. We observed that the percentages of memory CD8^+^ T cells and CD8^+^ TRM cells, particularly CD8^+^ CD103^-^ TRM cells, as well as plasmablasts in the MES T were significantly lower in patients with CRC showing T4 stage disease than those with T2-3 stage disease (**Figures 5A, B**). Moreover, our results showed that the percentage of memory CD4^+^ T cells in the MES T was higher in patients with distant metastasis (M1 stage CRC) than in those without distant metastasis (M0 stage CRC) (**Figure 5C**). Other lymphocytes were irrelevant to the tumor stage (data not shown). Therefore, our results indicate that decreased percentages of memory CD8^+^ T cells and plasmablasts in the MES T are associated with an increased depth of invasion of CRC, and increased percentage of memory CD4^+^ T cells is associated with distant metastasis of CRC.

### Increased Memory CD4^+^ T Cells Are Associated With High Preoperative Serum Carcinoembryonic Antigen Levels

**Figure 6:**
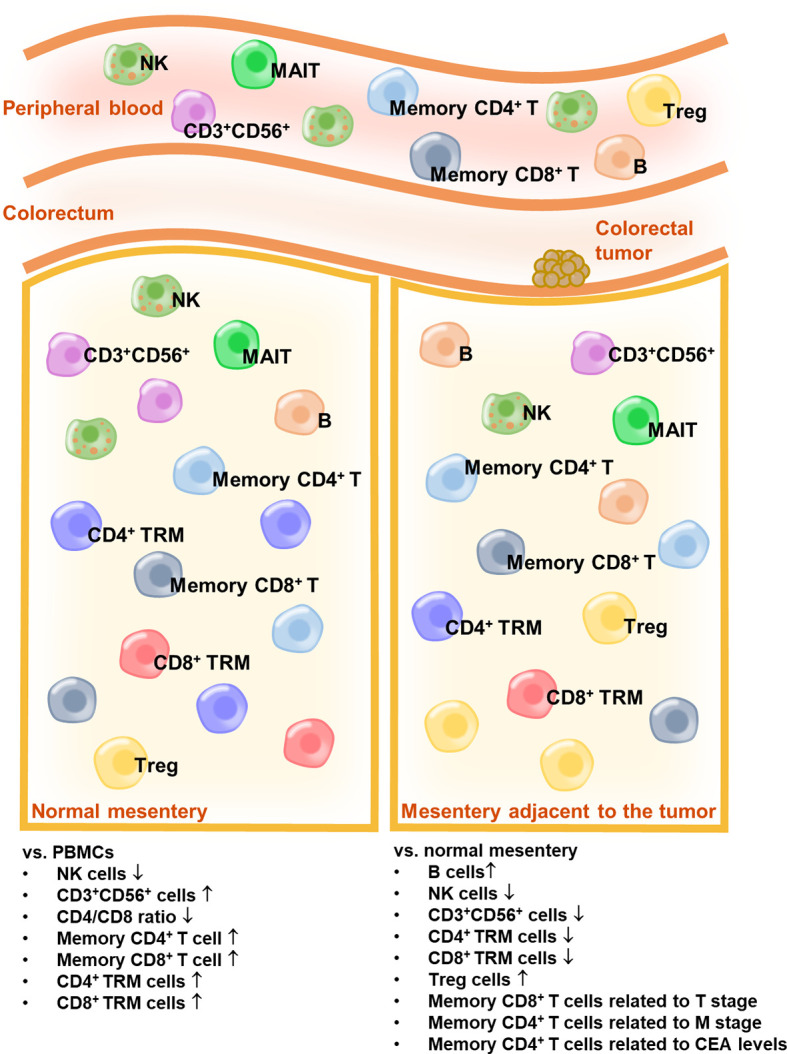
The schema of the immune landscapes of the PB, the MES N, and MES T in patients with CRC.

Serum CEA is hyperexpressed in patients with CRC and is widely used as a biomarker for CRC (31, 32). A preoperative serum CEA level ≥5 ng/mL is considered an independent prognostic factor for poor overall survival (33). Moreover, preoperative serum CEA elevation (≥5 ng/mL) is associated with a high recurrence rate (34). In our study, we observed a significantly high percentage of memory CD4^+^ T cells in the MES T of patients with CRC, who showed preoperative serum CEA levels ≥5 ng/mL (**Figure 5D**). Other lymphocytes were irrelevant to the preoperative serum CEA levels (data not shown). Overall, our data indicate that a high percentage of memory CD4^+^ T cells in the MES T is a predictor of distant metastasis of CRC and CRC recurrence. The schema of the immune landscapes of the PB, the MES N, and MES T in patients with CRC is displayed in **Figure 6**.

## Discussion

Following latest research in this field, the mesentery is currently classified as a distinct organ (13) that is histologically characterized by surface mesothelium, the connective tissue lattice, and adipocytes dispersed within the interstices of the lattice (13). However, the immune landscape of the mesentery remains unclear. Based on the findings of this study, we could successfully establish that the MES N shows a specific immune microenvironment that differs from that observed in the PB. We also observed that the immune cells detected in the MES N are primarily of lymphoid lineage. CRC can alter the mesenteric immune response to one which favors tumor promotion. Our study identified more specific and comprehensive lymphocyte populations in the mesentery, which provides a deeper understanding of the mesenteric immune microenvironment and its association with clinicopathological characteristics of patients with CRC.

Intestinal tumors can invade the contiguous mesentery. Studies have proved that the mesentery could serve as a potential site of residual tumors, leading to postoperative local CRC recurrence (6–8). Previous reports have characterized the mesenteric immune microenvironment with regard to macrophage infiltration (18); however, the lymphocyte subpopulations of the MES T remain unclear. Our study is the first to highlight that anti-tumor lymphocytes (NK and CD8^+^ TRM cells, specifically CD8^+^ CD103^-^ TRM cells) were decreased and immunosuppressive lymphocytes (Treg cells, specifically effector Treg cells) were increased in the MES T. These results indicate poor anti-tumor immunity in the MES T, which favors CRC development. Therefore, it can be inferred that the MES T may represent a component of the tumor microenvironment, and it is important to consider the potential role of the mesentery as a contributor to the development and progression of intestinal diseases.

Furthermore, our data indicate that memory CD8^+^ T cells, particularly CD8^+^ TRM cells and plasmablasts in the MES T are negatively correlated with the depth of invasion of CRC. Based on expression of high levels of TNF-α, IFN-γ, and granzyme B, CD8^+^ TRM cell infiltration in various human cancers is correlated with favorable clinical outcomes, and CD8^+^ TRM cells have emerged as a predictive marker of survival in several human epithelial cancers (35–40). These results suggest that mesenteric CD8^+^ TRM cells may play a protective role against CRC. Additionally, a high percentage of memory CD4^+^ T cells is a hallmark of adaptive immune memory (41). Our results indicate that enhanced expression of memory CD4^+^ T cells in the MES T is associated with distant metastasis of CRC, which could be attributable to the fact that CD4^+^ T cells are activated and differentiated into memory CD4^+^ T cells in the MES T in patients with distant metastasis. Moreover, our data show a significantly high percentage of memory CD4^+^ T cells in the MES T in patients with preoperative serum CEA levels ≥5 ng/mL. Serum CEA, which is a well-known marker of CRC, is hyperexpressed in patients with CRC, particularly in those showing CRC recurrence (31). High preoperative serum CEA levels suggest advanced disease with local or distant metastasis (42, 43). A cut-off level of 5 ng/mL is used to define CEA elevation; cancer-specific mortality rates are higher and prognosis is poorer in patients with CRC showing preoperative serum CEA levels ≥5 ng/mL (44). Taken together, our results indicate that memory CD4^+^ T cells may emerge as a useful predictive marker of distant metastasis of CRC and CRC recurrence, and this subpopulation of lymphocytes may serve as a potential target for the treatment of patients with CRC.

In addition to CRC, many intra-abdominal tumors, such as ovarian (45, 46), endometrial (47), gastric (48), and pancreatic cancer (49) show a predilection for peritoneal metastasis with poor prognosis. Moreover, recent studies have provided a wide range of evidence that describes visceral obesity as an important etiological contributor to cancer (50, 51). Therefore, further research on the mesenteric immune microenvironment is warranted considering its overall significance, not limited to CRC. Furthermore, regulation of the mesenteric immune microenvironment may be a useful therapeutic strategy for metastatic tumors. It is noteworthy that the clinical relevance of the mesentery is not confined to abdominal disease (52–54); the mesentery is the single greatest contributor to visceral adiposity (55), which plays an important role in the pathobiology of obesity, diabetes, and metabolic syndrome (56). It is equally important to focus on the involvement of the mesenteric immune microenvironment in these disorders.

In summary, we investigated the lymphocyte landscape of the mesentery and observed that it was correlated with CRC progression. However, the limitations of our study cannot be ignored; the mechanisms of how CRC affects the composition and phenotype of mesenteric lymphocytes and the long-term effects of mesenteric lymphocytes on the prognosis of patients with CRC remain unclear. Further investigations and large-scale studies that include long-term survival data are warranted to confirm the association between CRC and the mesenteric immune microenvironment.

## Data Availability Statement

The original contributions presented in the study are included in the article/**Supplementary Material**. Further inquiries can be directed to the corresponding authors.

## Ethics Statement

The studies involving human participants were reviewed and approved by Guangzhou First People’s Hospital Ethics Review Committee. The patients/participants provided their written informed consent to participate in this study. Written informed consent was obtained from the individual(s) for the publication of any potentially identifiable images or data included in this article.

## Author Contributions

Z-XL, JC, Z-BZ, and W-LL conceived and participated in the design of the study and revised the manuscript. The manuscript was written and revised by Z-XW and FW. Z-XW, FW, and Q-QL performed the experimental work. Z-XW and Q-QL collected the clinical data. LL, YY, and JL participated in data analysis. All authors contributed to the article and approved the submitted version.

## Funding

This work was supported by Program for Guangdong Introducing Innovative and Entrepreneurial Teams (2017ZT07S054), Guangdong Basic and Applied Basic Research Foundation (2020A1515010897), Special Clinical Technology Program of Guangzhou (2019TS52), Guangzhou Science Technology and Innovation Commission (201804010073), National Natural Science Foundation of China (81871943), Guangzhou Science Technology and Innovation Commission (201805010003), Outstanding Scholar Program of Bioland Laboratory (Guangzhou Regenerative Medicine and Health Guangdong Laboratory) (2018GZR110102001) and Guangdong Basic and Applied Basic Research Foundation (2021B1515420004).

## Conflict of Interest

The authors declare that the research was conducted in the absence of any commercial or financial relationships that could be construed as a potential conflict of interest.

## Publisher’s Note

All claims expressed in this article are solely those of the authors and do not necessarily represent those of their affiliated organizations, or those of the publisher, the editors and the reviewers. Any product that may be evaluated in this article, or claim that may be made by its manufacturer, is not guaranteed or endorsed by the publisher.
