# Supplementary material for: The Clinical Significance of Mesenteric Lymphocytes in Human Colorectal Cancer
Source: Front Oncol. 2021 Sep 16;11:685577. doi: 10.3389/fonc.2021.685577 (PMC8481834; doi:10.3389/fonc.2021.685577)
Supplement: Supplementary file 1 [file DataSheet_1.docx]

Supplementary Material

# Supplementary Figures

**
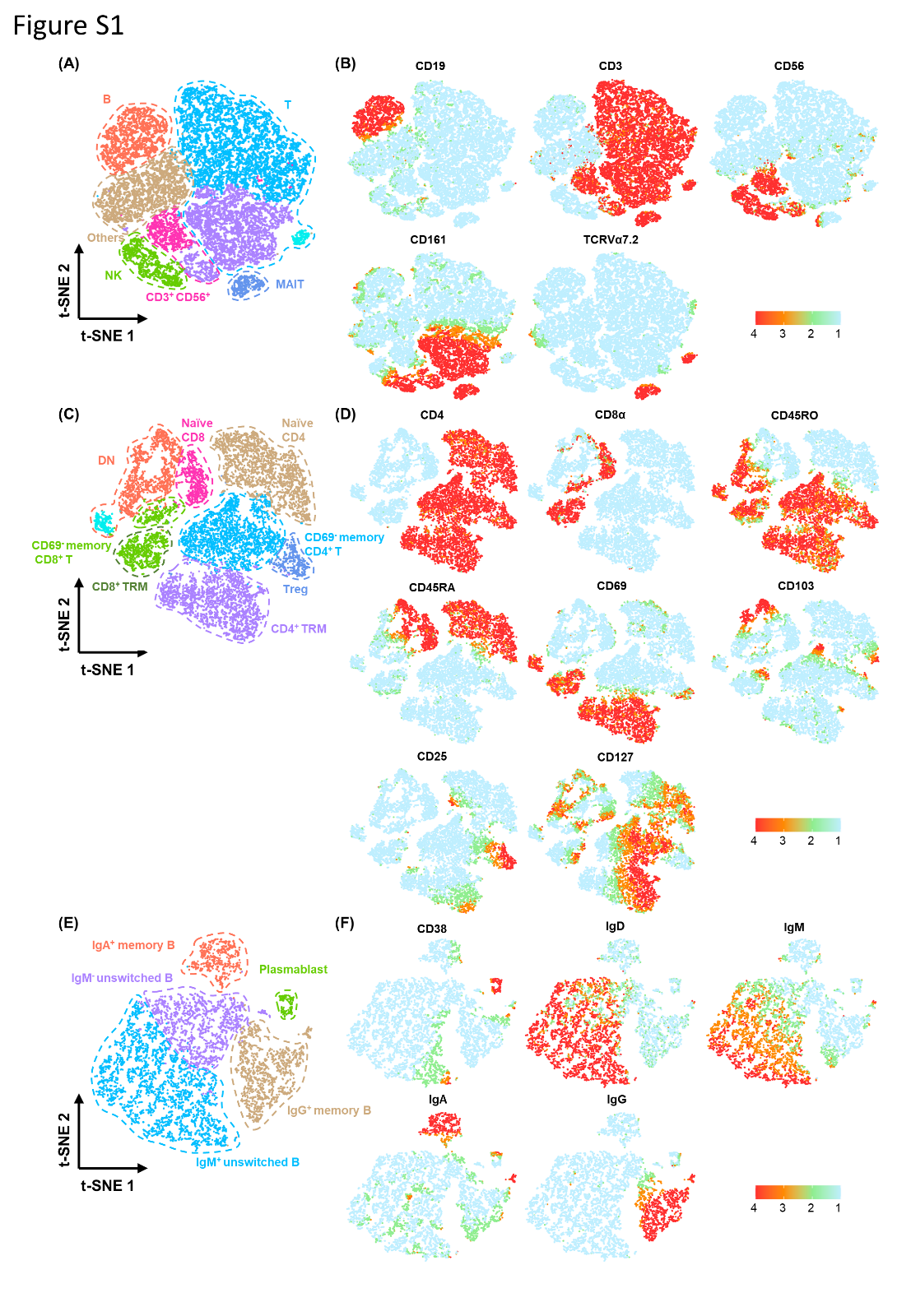
**

**Supplementary Figure 1.** T-SNE analysis of multiparameter flow cytometry data. (A) T-SNE plot of CD45^+^ MNCs in the PBMCs, MES N, and MES T with main subclusters indicated. (B) Feature plot of CD45^+^ MNCs markers. (C) T-SNE plot of T cells in the PBMCs, MES N, and MES T with main subsets indicated. (D) Feature plot of T cell markers. (E) T-SNE plot of B cells in the PBMCs, MES N, and MES T with main subsets indicated. (F) Feature plot of B cell markers.

**
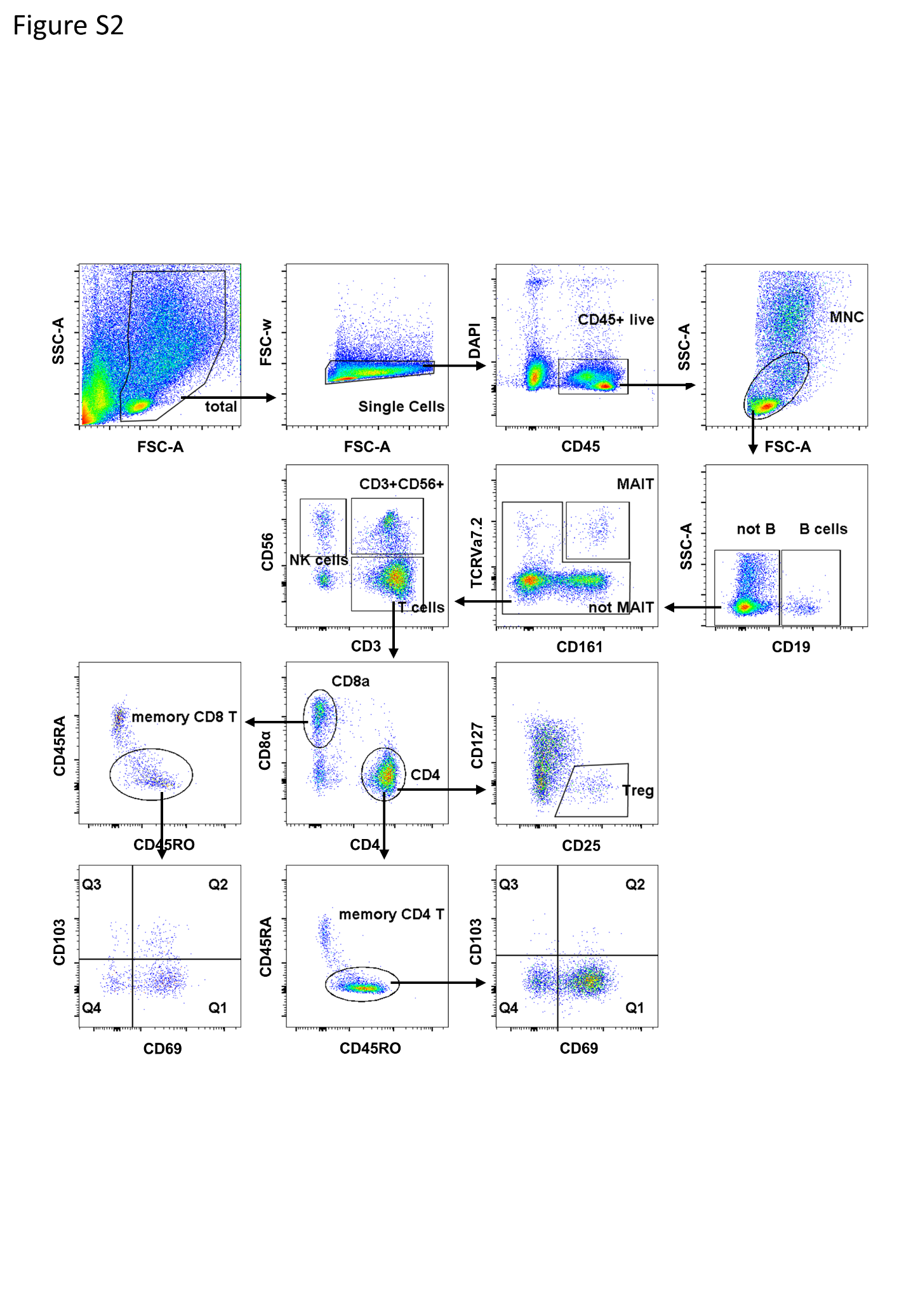
**

**Supplementary Figure 2.** Gating strategy of CD45^+^ MNCs subsets and T cell subsets.

**
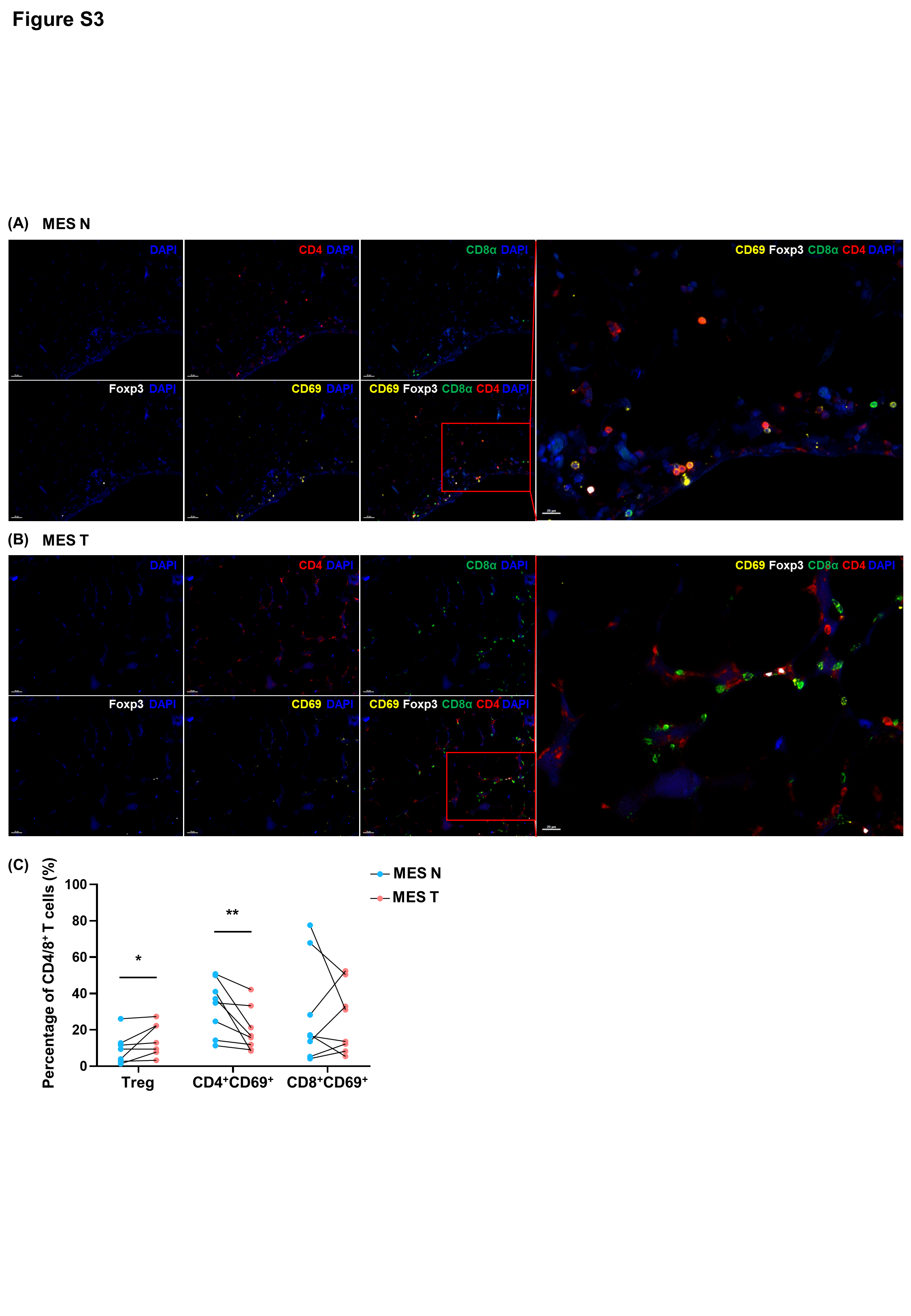
**

**Supplementary Figure 3.** The characteristic subsets of T cells in the FFPE MES N and the FFPE MES T from patients with CRC were stained by mIHC. (A) The FFPE MES N slides from patients with CRC were stained with DAPI (blue), CD4 (red), CD8 (green), Foxp3 (white) and CD69 (yellow). (B) The FFPE MES T slides from patients with CRC were stained with DAPI (blue), CD4 (red), CD8 (green), Foxp3 (white) and CD69 (yellow). (C) Percentages of Treg cells, CD4^+^ CD69^+^ T cells and CD8^+^ CD69^+^ T cells in the paired FFPE MES N and FFPE MES T from patients with CRC. (Each data point represents one sample. *p<0.05; **p<0.01.)

**
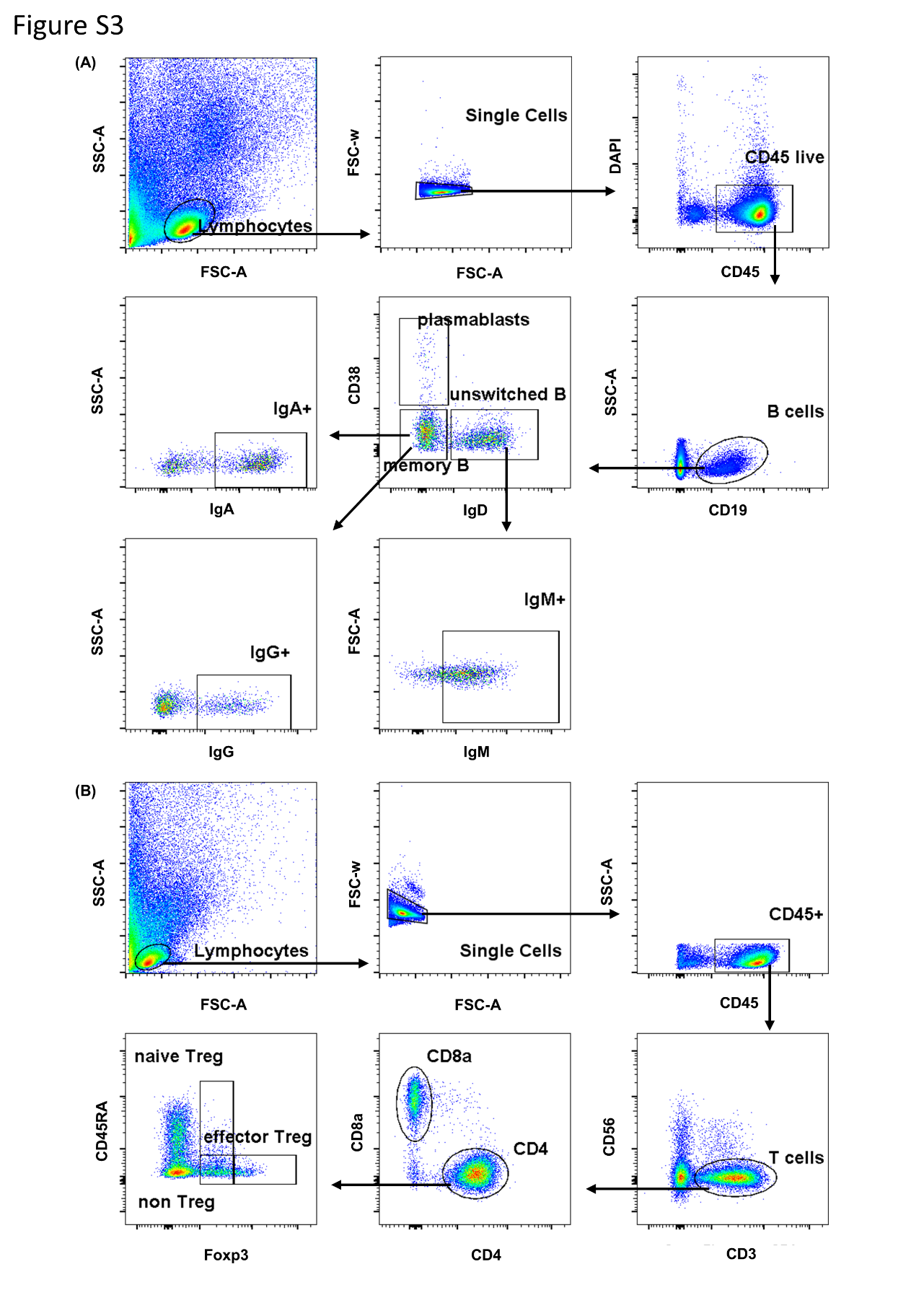
**

**Supplementary Figure 4.** Gating strategy of B cell subsets and Foxp3^+^ Treg cell subsets. (A) Gating strategy of B cell subsets. (B) Gating strategy of Foxp3^+^ Treg cell subsets.

**
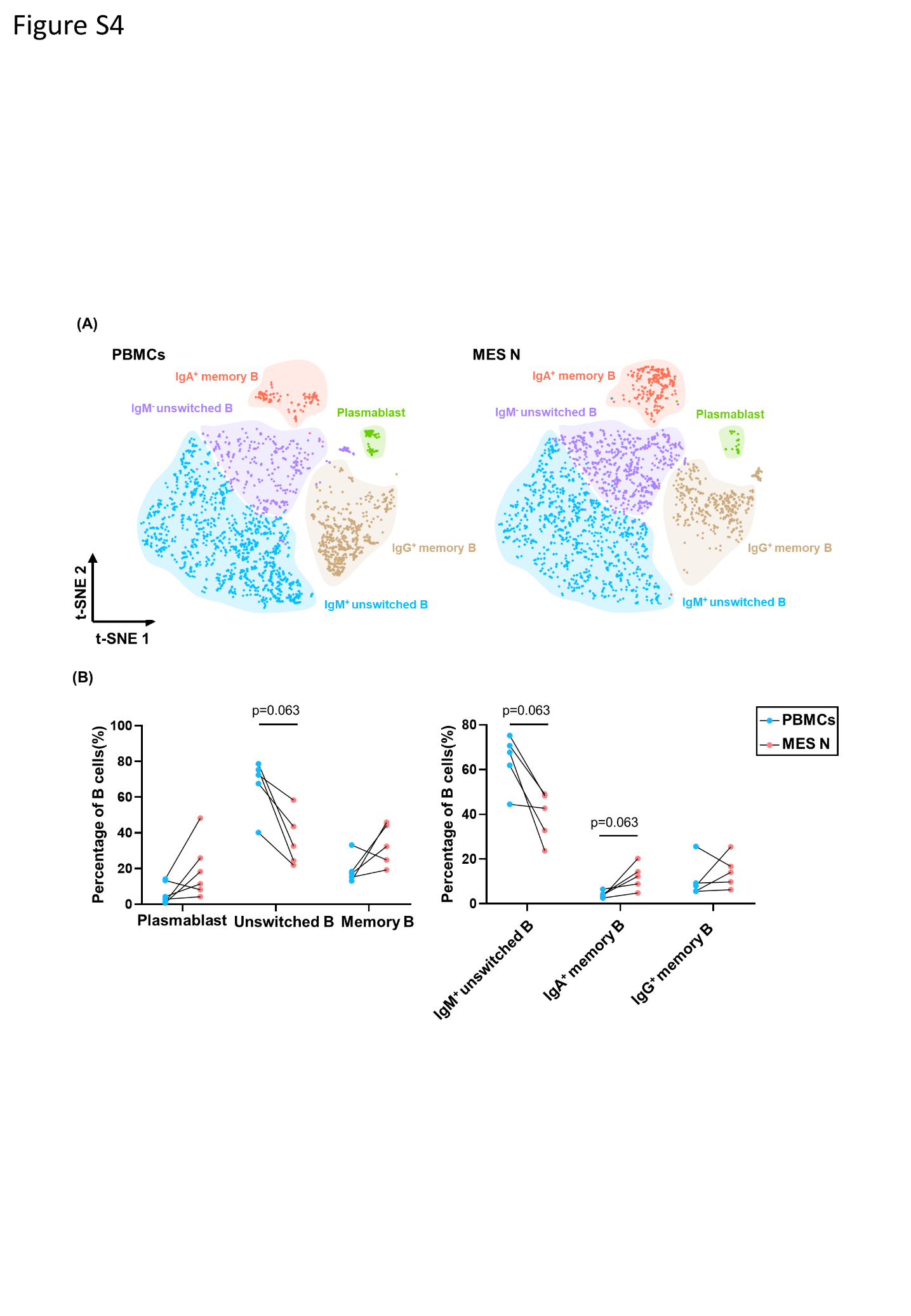
**

**Supplementary Figure 5.** Comparison of B cells in the PBMCs and MES N. (A) T-SNE plot of B cells in the PBMCs and MES N with main subclusters indicated. (B) Percentages of plasmablasts, unswitched B cells, memory B cells, IgM^+^ unswitched B cells, IgA^+^ memory B cells, and IgG^+^ memory B cells in the PBMCs and MES N.


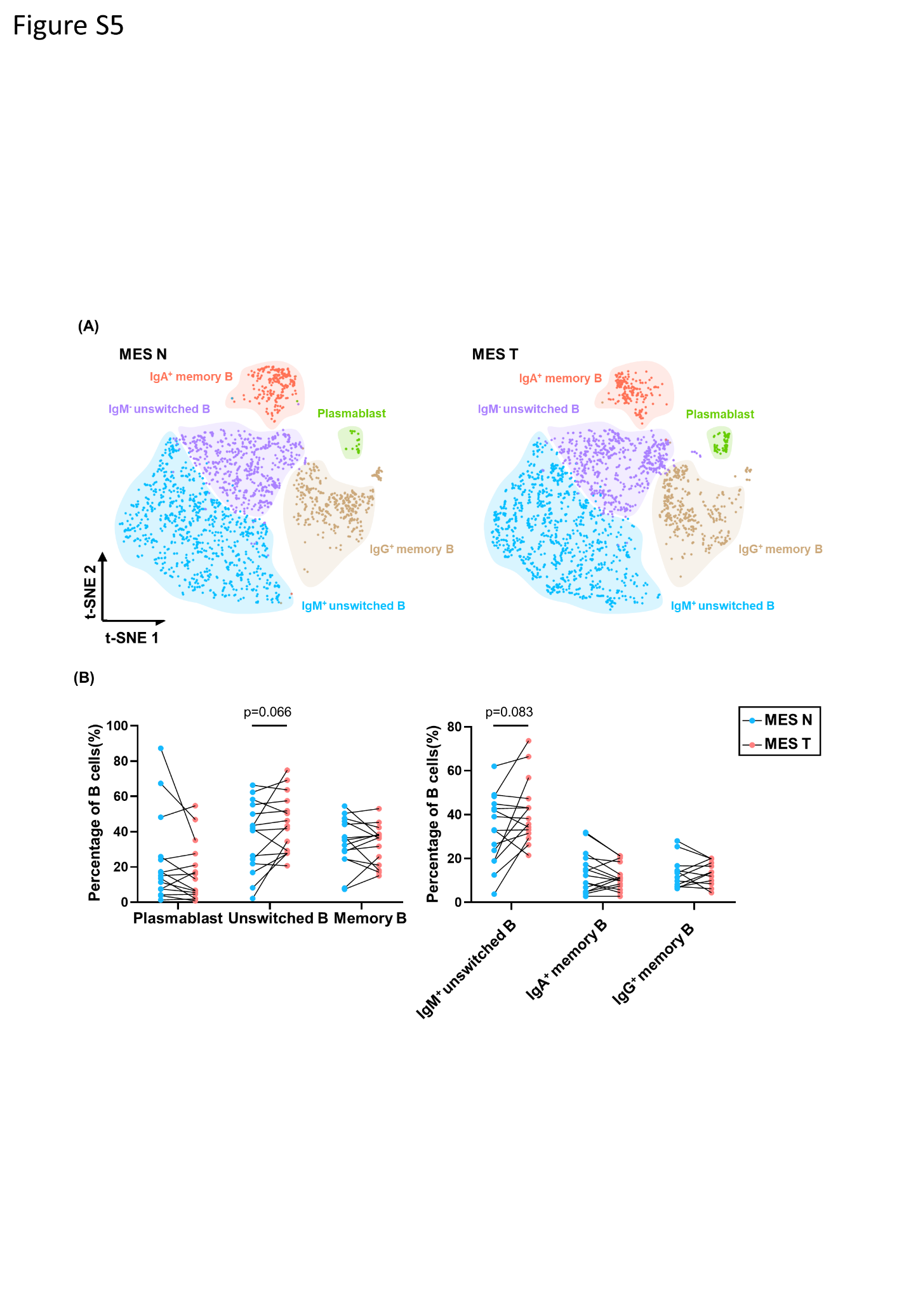


**Supplementary Figure 6.** Comparison of B cells in the MES N and MES T. (A) T-SNE plot of B cells in the MES N and MES T with main subclusters indicated. (B) Percentages of plasmablasts, unswitched B cells, memory B cells, IgM^+^ unswitched B cells, IgA^+^ memory B cells, and IgG^+^ memory B cells in the MES N and MES T.

# Supplementary Table

**Supplementary Table 1.** Analysis of gender and age between patients with CRC showing different TNM stages and preoperative serum CEA levels

| Characteristics | Gender, n (%) | | p | Age group, n (%) | | p |
| --- | --- | --- | --- | --- | --- | --- |
|  | Male | Female |  | <55 years | ≥55 years |  |
| **T stage** |  |  |  |  |  |  |
| 2-3 | 11 (64.7) | 6 (40.0) | 0.287 | 5 (55.6) | 12 (52.2) | 1.000 |
| 4 | 6 (35.3) | 9 (60.0) |  | 4 (44.4) | 11 (47.8) |  |
| **N stage** |  |  |  |  |  |  |
| 0 | 8 (47.1) | 9 (60.0) | 0.502 | 3 (33.3) | 14 (60.9) | 0.243 |
| 1-2 | 9 (52.9) | 6 (40.0) |  | 6 (66.7) | 9 (39.1) |  |
| **M stage** |  |  |  |  |  |  |
| 0 | 11 (84.6) | 6 (66.7) | 0.609 | 6 (75.0) | 11 (78.6) | 1.000 |
| 1 | 2 (15.4) | 3 (33.3) |  | 2 (25.0) | 3 (21.4) |  |
| **Preoperative**  **serum CEA level (ng/mL)** |  |  |  |  |  |  |
| <5 | 13 (76.5) | 8 (53.3) | 0.266 | 7 (77.8) | 14 (60.9) | 0.441 |
| ≥5 | 4 (23.5) | 7 (46.7) |  | 2 (22.2) | 9 (39.1) |  |

^a^Abbreviation: CEA, carcinoembryonic antigen.
